# Humoral Immune Responses to the Live-Attenuated African Swine Fever Vaccine Candidate VNUA-ASFV-LAV2 Reveal Potential Serological Markers for Vaccine Monitoring and DIVA Surveillance

**DOI:** 10.3390/pathogens15101005

**Published:** 2026-09-22

**Authors:** Lihua Wang, Quang Lam Truong, Yuzhen Li, Rachel Madera, Aidan Craig, Nina Muro, Xirui Zhang, Danqin Li, Limeng Cai, Jamie Retallick, Franco Matias Ferreyra, Lan Thi Nguyen, Eun-Ju Sohn, Jishu Shi

**Affiliations:** 1 Center on Biologics Development and Evaluation, Department of Anatomy and Physiology, College of Veterinary Medicine, Kansas State University, Manhattan, KS 66506, USA; yuzhen@ksu.edu (Y.L.); rachelmadera@ksu.edu (R.M.); aidan3@ksu.edu (A.C.); nina14@ksu.edu (N.M.); xzr92@missouri.edu (X.Z.); danqinli@ksu.edu (D.L.); lmcai98@ksu.edu (L.C.); 2 Key Laboratory of Veterinary Biotechnology, Faculty of Veterinary Medicine, Vietnam National University of Agriculture, Gia Lam, Ha Noi 12406, Vietnam; tqlam@vnua.edu.vn (Q.L.T.); nguyenlan@vnua.edu.vn (L.T.N.); 3 College of Veterinary Medicine, University of Missouri, Columbia, MO 65211, USA; 4 Department of Diagnostic Medicine and Pathobiology, College of Veterinary Medicine, Kansas State University, Manhattan, KS 66506, USA; retallick@ksu.edu (J.R.); francomf@ksu.edu (F.M.F.); 5 BioApplications Inc., Pohang-si 37668, Republic of Korea; ejsohn@bioapp.co.kr

**Keywords:** African swine fever, live-attenuated virus, vaccine, humoral immune response, ELISA, diagnostic, DIVA

## Abstract

African swine fever (ASF) is a highly contagious and often fatal disease of swine that causes substantial economic losses worldwide. Despite extensive efforts, the lack of safe and effective vaccines remains a major obstacle to ASF control. We previously developed a cell-adapted live-attenuated virus (LAV) vaccine candidate, VNUA-ASFV-LAV2, through serial passage of a highly virulent genotype II ASF virus (ASFV) in cell culture. VNUA-ASFV-LAV2 demonstrated favorable safety and protective efficacy against contemporary epidemic genotype II ASFV. In this study, we systematically evaluated antibody responses in pigs immunized with VNUA-ASFV-LAV2. Fourteen indirect ELISAs were developed using ASFV structural, nonstructural, and previously uncharacterized proteins associated with the viral outer envelope, capsid, inner envelope, and replication functions. Serum samples collected from vaccinated and challenged pigs were used to assess the performance of these assays. The results demonstrated that p17-, p22-, p30-, pE199L-, and pB602L-based ELISAs have strong potential for monitoring antibody responses in pigs vaccinated with VNUA-ASFV-LAV2. Among these assays, p22- and p30-based ELISAs enabled early detection of ASFV-specific antibodies in both vaccinated (as early as 7–14 days post vaccination) and wild-type ASFV-infected pigs (17 days post infection). Notably, the pF317L-based ELISA showed potential to differentiate wild-type ASFV-infected pigs from VNUA-ASFV-LAV2-vaccinated pigs. Further diagnostic validation using larger, independently characterized serum panels representing diverse ASFV strains, vaccination statuses, and epidemiological settings is needed to establish its sensitivity, specificity, reproducibility, and diagnostic accuracy. These findings provide the first comprehensive analysis of humoral immune responses elicited by VNUA-ASFV-LAV2 in pigs and identify potential serological markers for vaccine monitoring and DIVA surveillance, supporting future field applications and commercialization of this vaccine candidate.

## 1. Introduction

African swine fever (ASF) poses a severe threat to the global swine industry. Classified as a notifiable disease, it requires mandatory reporting to the World Organization for Animal Health (WOAH) [1]. This highly contagious and severe hemorrhagic transboundary viral disease, with up to a 100% mortality rate, causes tremendous socio-economic losses worldwide [2]. ASF was first reported in Africa in the 1920s and emerged in Europe in the 1960s [3,4]. Since 2007, ASF has spread steadily across Eastern European and neighboring countries. The situation escalated dramatically in August 2018 with ASF’s confirmation in China. The disease then rapidly spread to numerous pork-producing nations across Asia [5]. In 2019–2020, ASF reached Oceania, affecting Timor-Leste and Papua New Guinea [6]. In 2021, ASF spread to the Dominican Republic and Haiti, which are both dangerously close to United States [7]. More recently, outbreaks have been reported in additional European countries [8], underscoring the continued global expansion of ASF and highlighting the challenges associated with ASF control.

The causative agent, African Swine Fever Virus (ASFV), is a large, enveloped virus belonging to the *Asfarviridae* family and *Asfivirus* genus. Its complex genome consists of a double-stranded DNA (dsDNA) molecule ranging from 170 to 193 kilobase pairs (kbp) in size that encodes more than 150 proteins [9]. A total of 24 ASFV genotypes (I-XXIV) have been described based on the ASFV p72 major capsid protein gene (*B646L*) [10]. While all genotypes circulate in Africa [11], genotype II has been responsible for the ongoing transcontinental epidemic since 2007. More recently, recombinant genotype I/II ASFV strains have emerged in Asia and Russia [12,13,14], further complicating disease control efforts.

The lack of widely available, safe, and effective vaccines remains the greatest obstacle to preventing and controlling ASF. Consequently, current control strategies rely heavily on surveillance, rapid diagnosis, movement restrictions, culling of infected animals, and strict biosecurity measures. Over the past several decades, multiple vaccine platforms, including inactivated, subunit, DNA, mRNA, viral-vectored, and live-attenuated virus (LAV) vaccines, have been investigated. Inactivated vaccines, while exhibiting favorable safety profiles, have consistently shown poor efficacy, primarily due to inadequate functional antigen presentation, epitope damage during inactivation, weak cellular immune activation, and the limited effectiveness of conventional adjuvants [15,16]. Subunit, DNA, and vectored vaccines targeting selected viral proteins are capable of eliciting measurable immune responses, yet they frequently fail to confer sterile or long-term protection [17,18]. In contrast, LAVs have consistently demonstrated superior protective efficacy and are currently considered the most promising vaccine platform for ASF [19,20,21]. However, the widespread implementation of ASFV LAVs faces important challenges. In particular, reliable assays for monitoring vaccine-induced immune responses remain limited, and few diagnostic tools are available to differentiate infected from vaccinated animals (DIVA). The development of sensitive serological assays and DIVA-compatible diagnostic tools will be essential for vaccine evaluation, field implementation, disease surveillance, and regulatory approval.

In response to the ongoing ASF epidemic, our group has focused on the development of ASF vaccines and companion diagnostic tools since 2019 [18,21,22,23]. As part of these efforts, we developed a cell-adapted live-attenuated ASFV vaccine candidate, VNUA-ASFV-LAV2, through serial passaging of a virulent genotype II ASFV field isolate in primary porcine alveolar macrophages (PAMs) and the immortalized porcine alveolar macrophage cell line 3D4/21 [22]. VNUA-ASFV-LAV2 replicates efficiently in both cell types and confers complete protection against challenge with contemporary genotype II ASFV field isolates. Vaccinated pigs remained clinically healthy, with no observed signs of anorexia, depression, fever, purple skin discoloration, staggering gait, diarrhea, or coughing, throughout the 28-day post-immunization observation period and the subsequent 28-day challenge period with VNUA-ASFV-05L1, a representative contemporary genotype II ASFV strain. VNUA-ASFV-LAV2 was cleared from blood by 28 days post-immunization (DPI) and from feces and oral fluids by 17 DPI, while vaccinated pigs remained protected against challenge. Post-mortem examination revealed no apparent pathological lesions in major organs, and VNUA-ASFV-LAV2 was undetectable in these tissues by real-time quantitative PCR (qPCR). Collectively, these findings support VNUA-ASFV-LAV2 as a promising LAV candidate with favorable safety and protective efficacy profiles [22].

The objectives of the present study are to identify ASFV proteins that elicit robust antibody responses following VNUA-ASFV-LAV2 vaccination and to evaluate their utility as serological markers for vaccine monitoring and DIVA-based surveillance. To this end, we developed a panel of indirect ELISAs based on ASFV structural, nonstructural, and previously uncharacterized proteins and evaluated their performance using serum samples collected from vaccinated and challenged pigs. Our findings provide valuable insights into the serological responses induced by VNUA-ASFV-LAV2 and identify promising protein-based indirect ELISAs (iELISAs) for monitoring vaccine-induced immunity and differentiating wild-type ASFV-infected from vaccinated pigs.

## 2. Materials and Methods

### 2.1. Protein Expression and Purification

Protein expression in *Nicotiana benthamiana* and the baculovirus expression system was performed as previously described [24,25]. Briefly, DNA sequences encoding ASFV Georgia 2007/1 (GenBank accession no. FR682468.2) proteins listed in Table 1 were codon optimized for expression in *N. benthamiana* or the baculovirus system and synthesized by GenScript (Piscataway, NJ, USA). A C-terminal six-histidine (6×His) tag was fused to each recombinant protein to facilitate purification. For protein expression in *N. benthamiana,* genes were transformed into Agrobacterium tumefaciens strain LBA4404 before vacuum infiltration of 5-week-old *N. benthamiana* plants. Infiltrated leaves were harvested after 4 days, ground, and resuspended in extraction buffer for subsequent recombinant protein purification. For baculovirus expression, the genes were cloned into the pFastBac™ 1 vector and transformed into DH10Bac™ *Escherichia coli* cells (Thermo Scientific, Waltham, MA, USA) to generate recombinant bacmids. Bacmid DNA was transfected into Sf9 insect cells to produce recombinant baculovirus, which was amplified through three serial passages. At passage 3, culture supernatants were harvested and clarified by centrifugation at 500× *g* for 5 min. The resulting baculovirus stocks were used to infect High Five™ insect cells for recombinant protein expression. Recombinant proteins were purified by immobilized metal affinity chromatography using Ni-NTA agarose (Thermo Fisher Scientific) according to the manufacturer’s instructions. Purified proteins were concentrated using Amicon Ultra centrifugal filter units (Millipore, Billerica, MA, USA), and protein concentrations were determined using a BCA Protein Assay Kit (Thermo Fisher Scientific).

### 2.2. Porcine Serum Samples

The collection and experimental use of porcine serum samples were performed as previously described [23]. Briefly, serum samples were collected from conventional Large White × Duroc specific-pathogen-free (SPF) weaned piglets experimentally vaccinated with VNUA-ASFV-LAV2 and/or challenged with the virulent ASFV strain VNUA-ASFV-05L1. Samples were maintained at Kansas State University (KSU) and used to evaluate antibody responses against ASFV proteins. All sample handling and laboratory procedures were conducted in Class III biosafety cabinets within the designated ASFV biosafety level 3 (BSL-3) laboratory at the Biosecurity Research Institute (BRI), KSU. The serum panel comprised the following categories:

(i) ASFV-negative sera: Serum samples collected from pigs (n = 30) inoculated with phosphate-buffered saline (PBS, pH 7.4; Thermo Scientific, Bridgewater, NJ, USA).
(ii) Vaccinated pig sera: Serum samples from pigs (n = 4) vaccinated with VNUA-ASFV-LAV2 (10^3^ HAD_50_/pig), collected at 7, 14, 21, and 28 days post vaccination (DPV).
(iii) Vaccinated and challenged pig sera: Serum samples from pigs (n = 4) vaccinated with VNUA-ASFV-LAV2 (10^3^ HAD_50_/pig) and subsequently challenged with VNUA-ASFV-05L1 (10 HAD_50_/pig). Samples were collected at 0, 7, 14, 21, 28 DPV and 3, 7, 10, 14, 17, and 21 days post challenge (DPC).
(iv) Dose-escalation vaccinated and challenged pig sera: Serum samples from pigs (n = 6/group) vaccinated with VNUA-ASFV-LAV2 at doses of 10^2^ (Group 1), 10^3^ (Group 2), and 10^4^ HAD_50_/pig (Group 3), followed by challenge with VNUA-ASFV-05L1 (10 HAD_50_/pig). Samples were collected at 0, 7, 14, 21, 28 DPV and 3, 7, 10, 14, and 17–21 DPC.
(v) Wild-type ASFV-challenged pig sera: Serum samples from pigs (n = 3) challenged with VNUA-ASFV-05L1 (10 HAD_50_/pig) only, collected from DPC 8 to DPC 21.

### 2.3. Indirect ELISA (iELISA)

The iELISA was performed as previously described [23]. Briefly, optimal antigen concentrations were determined by checkerboard titration using positive and negative reference sera, followed by optimization of serum dilution using two-fold serial dilutions. The optimized iELISAs were performed in Corning^®^ 96-well clear, flat-bottom, high-binding polystyrene microplates (Corning, NY, USA).

Plates were coated overnight at 4 °C with 100 μL/well of recombinant ASFV protein (1 μg/mL) diluted in phosphate-buffered saline (PBS; pH 7.4; Thermo Scientific, Bridgewater, NJ, USA). Plates were then washed three times with PBS containing 0.05% Tween 20 (PBST) and blocked with 300 μL/well of 5% skim milk in PBS at 37 °C for 1 h. After washing, 100 μL/well of porcine serum diluted 1:500 was added and incubated at 37 °C for 1 h. Plates were washed again and incubated with 100 μL/well of rabbit anti-porcine IgG F(ab′)_2_-HRP secondary antibody (1:10,000; Novus Biologicals, Centennial, CO, USA) at 37 °C for 1 h. After a final washing step, 100 μL/well of 3,3′,5,5′-tetramethylbenzidine (TMB) substrate (Invitrogen, Carlsbad, CA, USA) was added and incubated for 10 min. The reaction was stopped by adding 100 μL/well of 1 M sulfuric acid, and absorbance was measured at 450 nm (OD_450_) using a SpectraMax microplate reader (Molecular Devices, San Jose, CA, USA).

Each experiment included independent biological replicates, and each serum sample was tested in duplicate wells. The cutoff value for each iELISA was determined using sera from PBS-inoculated pigs (serum panel category i). A sample was considered positive when its OD_450_ value exceeded the mean OD_450_ of the negative-control sera plus three standard deviations (mean + 3SD). Sensitivity and specificity were calculated using the web-based MedCalc (Version 23.7.1) diagnostic test calculator (https://www.medcalc.org/calc/diagnostic_test.php, accessed on 14 September 2026).

## 3. Results

### 3.1. Optimization of Protein Coating Concentration and Serum Dilution for iELISAs

Checkerboard titration was conducted to optimize the protein coating concentration and serum dilution for the iELISAs with positive and negative reference porcine serum samples. Negative reference sera were obtained from PBS-inoculated pigs (serum panel category i), whereas positive reference sera were obtained from VNUA-ASFV-LAV2-vaccinated pigs at 28 DPV (serum panel category ii) and wild-type VNUA-ASFV-05L1-challenged pigs at DPC21 (serum panel category v). Purified proteins were coated onto microplates at two-fold serial dilutions ranging from 32 to 0.016 μg/mL (100 μL/well). A protein concentration of 1 μg/mL was selected for subsequent assays, as it produced the greatest OD difference between positive and negative reference sera and yielded a high signal-to-noise ratio. The optimal serum dilution was subsequently determined using plates coated with 1 μg/mL protein and testing two-fold serial dilutions of reference sera beginning at 1:5. A serum dilution of 1:500 was selected because it provided strong discrimination between positive and negative samples while minimizing background reactivity. These optimized assay conditions were applied in all subsequent iELISA experiments.

### 3.2. iELISAs Suitable for Monitoring Antibody Responses Following VNUA-ASFV-LAV2 Vaccination

A total of 14 iELISAs were developed and evaluated using serum panels i–v described in Section 2.2. The ASFV proteins selected as coating antigens represented different functional categories (Table 1) and were chosen based on their reported immunogenicity and their use as potential subunit vaccine targets. We evaluated their ability to test antibody responses following VNUA-ASFV-LAV2 vaccination with serum panel iii samples. The results showed that among these assays, iELISAs based on p17, p22, p30, pE199L, and pB602L demonstrated robust detection of vaccine-induced antibody responses, identifying seroconversion in VNUA-ASFV-LAV2-vaccinated pigs between 14 and 21 DPV. However, the p54-based iELISA detected specific anti-p54 antibodies only from DPV28 onward (Figure 1).

The remaining iELISAs, including those utilizing CD2v, C-type lectin, p15, p35, p72, pMGF505-5R, pF317L, and pI177L as coating antigens, showed limited ability for monitoring vaccination responses. Specifically, the C-type lectin-based iELISA detected seroconversion at DPC7, while the CD2v-, p15-, and p35-based iELISAs turned positive at DPC15. In contrast, iELISAs based on p72, pMGF505-5R, pF317L, and pI177L remained negative across all sampling time points (Table 2).

These findings demonstrate that p17-, p22-, p30-, pE199L-, and pB602L-based iELISAs are promising assays for serological monitoring of VNUA-ASFV-LAV2 vaccination. In contrast, the remaining protein-based iELISAs exhibited limited sensitivity in detecting antibody responses elicited by either VNUA-ASFV-LAV2 vaccination or wild-type ASFV infection.

### 3.3. iELISAs Suitable for Early Detection of Antibody Responses Following Vaccination and ASFV Infection

To confirm the ability of the p22- and p30-based iELISAs to detect early immune responses following vaccination or ASFV infection, we evaluated these assays using additional serum samples. In the experiments above, both assays detected ASFV-specific antibodies as early as 14 DPV (Table 2). We further evaluated these assays using serum samples from pigs vaccinated with VNUA-ASFV-LAV2 at doses of 10^2^, 10^3^, or 10^4^ HAD_50_/pig (serum panel category iv), as well as sera from pigs challenged with wild-type ASFV (serum panel category v). The p22- and p30-based iELISAs successfully detected ASFV-specific antibody responses between DPV7 and DPV14 across all vaccination doses, ranging from the lowest dose (10^2^ HAD_50_/pig) to the highest dose (10^4^ HAD_50_/pig), demonstrating their suitability for early monitoring of vaccine-induced humoral immune responses (Figure 2). During the vaccination phase, pigs receiving the highest vaccine dose (10^4^ HAD_50_/pig) generally developed stronger antibody responses than those receiving lower doses. However, following challenge with virulent ASFV, antibody levels did not show a clear dose-dependent relationship, suggesting that post-challenge immune responses were influenced by infection dynamics rather than the initial vaccination dose. In addition, both p22- and p30-based iELISAs effectively detected antibody responses induced by wild-type ASFV infection. Positive antibody responses were detected as early as DPC17 in pigs challenged with wild-type ASFV (10 HAD_50_/pig). In contrast, the other iELISAs evaluated in this study failed to detect detectable antibody responses at the same time point.

### 3.4. Identification of a Potential DIVA iELISA for VNUA-ASFV-LAV2 Vaccination

All 14 iELISAs were evaluated using serum samples from pigs vaccinated with VNUA-ASFV-LAV2 (10^2^–10^4^ HAD_50_/pig), vaccinated and subsequently challenged with VNUA-ASFV-05L1 (10 HAD_50_/pig), or infected with wild-type ASFV only (10 HAD_50_/pig) to identify assays capable of differentiating infected from vaccinated animals. Of the 14 assays tested, 13 failed to demonstrate DIVA capability, as they showed either positive or negative profiles in both VNUA-ASFV-LAV2-vaccinated and wild-type ASFV-infected pig serum samples. In contrast, the pF317L-based iELISA consistently remained negative in VNUA-ASFV-LAV2-vaccinated pigs through 28 DPV, resulting in an observed specificity of 100% (95% confidence interval: 95.49% to 100%; calculated from 80 serum samples collected across 20 individual pigs). The assay showed a coefficient of variation (CV) of 22.26%, reflecting the dispersion of OD values across the vaccinated sample set. This higher relative CV in the vaccinated panel is attributable to low baseline OD values near the negative control background, where minor absolute fluctuations yield higher relative percentage CVs; however, all individual values remained well below the diagnostic cutoff threshold. Conversely, following challenge or wild-type infection, robust antibody responses generated higher mean OD values, resulting in a lower relative CV of 15.77% across the challenged/infected sample set. Specifically, the assay detected pF317L-specific antibodies in vaccinated pigs following challenge with VNUA-ASFV-05L1, becoming positive between DPC17 and DPC21, and in pigs infected with wild-type ASFV alone from DPC8 to DPC21 (Figure 3), yielding an observed sensitivity of 100% (95% confidence interval: 59.04% to 100%; calculated from 7 serum samples from 7 individual pigs). Note that these binomial confidence intervals are sample-based and are not adjusted for within-animal clustering across repeated time points. These findings indicate that antibodies against pF317L are induced following wild-type ASFV infection but not after VNUA-ASFV-LAV2 vaccination, supporting the potential of pF317L as a DIVA serological marker.

## 4. Discussion

Serological detection of ASFV-specific antibodies in peripheral blood is a key component of ASF surveillance and vaccine monitoring, as recommended by the World Organisation for Animal Health (WOAH) [41]. Among serological methods, iELISA is one of the most widely used assays for detecting antigen-specific antibodies because of its high sensitivity, flexibility, and cost-effectiveness [42]. In this study, we developed a panel of iELISAs using 14 recombinant ASFV proteins to characterize humoral immune responses induced by the LAV vaccine candidate VNUA-ASFV-LAV2. This vaccine candidate demonstrated favorable protective efficacy and safety in pigs during the 28-day post-immunization period and the subsequent 28-day challenge period following exposure to the virulent parental strain, VNUA-ASFV-05L1. However, potential late-onset and field-condition adverse reactions were not assessed in the present study and warrant further investigation.

The 14 antigens were selected based on their biological functions and reported immunogenicity (Table 1). CD2v and C-type lectin-like proteins are viral envelope proteins involved in hemadsorption, cell adhesion, and immune modulation [26,27,28,29]. p17, p22, p30, p54, p72, and pE199L are structural proteins that participate in virus entry, capsid assembly, and virion morphogenesis [28,29,30,31,32,33,34,35]. p15 and p35 are core shell proteins involved in viral transcription, core assembly, and replication [36,37], whereas pB602L and pMGF505-5R are nonstructural proteins associated with virus replication and host adaptation [38,39]. We also included pF317L, an immunomodulatory protein implicated in innate immune evasion, and pI177L, a virulence-associated protein whose deletion has been exploited in the development of ASFV LAV vaccines [19,20,40].

Among the evaluated proteins, p17-, p22-, p30-, pE199L-, and pB602L-based iELISAs efficiently detected vaccine-induced antibodies between 14 and 21 DPV, consistent with previous reports demonstrating that these proteins are highly immunogenic during ASFV infection [43]. Notably, the p22- and p30-based iELISAs detected antibody responses as early as 7–14 DPV across all vaccine doses tested (10^2^–10^4^ HAD_50_/pig), consistent with previous studies showing that ASFV-specific antibodies can be detected within approximately 7–14 days following live-attenuated vaccination [43,44]. These findings indicate that p22 and p30 may be particularly useful serological markers for early monitoring of humoral responses following VNUA-ASFV-LAV2 vaccination (Figure 2). In contrast, the pMGF505-5R- and pI177L-based iELISAs did not detect vaccine-induced antibodies (Table 2). The CD2v-, C-type lectin-, p15-, and p35-based iELISAs did not detect vaccine-induced antibodies through 28 DPV but became positive after challenge (7–15 DPC), suggesting that these antigens induce relatively weak or delayed antibody responses following vaccination and therefore have limited sensitivity for serological monitoring.

Unexpectedly, the p72-based iELISA did not detect antigen-specific antibodies at any of the sampling time points. This finding may reflect limitations in the antigen used in the assay, including the possibility that the recombinant p72 did not fully reproduce the native conformation or oligomeric structure of the viral capsid protein in our protein expression system. In addition, factors related to antigen preparation, coating conditions, and assay optimization may have affected the ability of the iELISA to detect p72-specific antibodies. Differences in antibody responses induced by vaccination and subsequent challenge compared with those elicited by primary infection with virulent ASFV may also contribute to the absence of detectable p72-specific antibodies in this study. Further studies are needed to clarify the mechanisms underlying this observation.

Among all proteins evaluated, pF317L showed potential to differentiate wild-type ASFV-infected pigs from VNUA-ASFV-LAV2-vaccinated pigs. pF317L is a late-expressed ASFV immunomodulatory protein that suppresses NF-κB-mediated innate immune responses and elicits both humoral and cellular immune responses [40,45]. In the present study, the pF317L-based iELISA remained negative in all VNUA-ASFV-LAV2-vaccinated pigs but became positive in pigs challenged with VNUA-ASFV-05L1 and in pigs infected with wild-type ASFV alone (Figure 3). These findings indicate that pF317L-specific antibodies are induced following wild-type ASFV infection but not after vaccination with VNUA-ASFV-LAV2. Whole-genome sequencing analysis showed that, compared with the wild-type parental virus VNUA-ASFV-05L1 (GenBank accession number MW465755.1), the *F317L* sequence was identical between the parental wild-type ASFV and VNUA-ASFV-LAV2. Examination of the genomic regions surrounding *F317L* revealed two nucleotide mutations [22]. The first is a single-nucleotide deletion at genome position 140,235 within *A104R*, leading to a premature stop codon that could potentially alter host immune modulation [46]. The second is a single-nucleotide substitution at position 133,757, causing a methionine-to-threonine swap in the adjacent *A179L* gene. Whether these mutations impair pF317L expression or function and drive the loss of pF317L-specific antibody responses post-vaccination with VNUA-ASFV-LAV2 remains to be determined. Further mechanistic studies, including evaluating pF317L expression levels in infected macrophages via Western blot and proteomics, are planned for a follow-up study and will be presented in a subsequent publication.

One limitation of this study is the relatively small number of serum samples analyzed, particularly for the evaluation of the pF317L-based iELISA. This assay has potential utility for post-marketing pharmacovigilance by enabling serological differentiation of vaccine exposure from intercurrent wild-type ASFV infection in vaccinated herds, thereby facilitating investigation of vaccine-associated events. To further validate diagnostic performance of the developed iELISAs, we are collaborating with partners in Vietnam to expand the collection of field serum samples, with particular emphasis on the pF317L-based iELISA. Future studies will include larger, independently characterized serum panels representing diverse ASFV strains, vaccination statuses, and epidemiological settings to robustly assess assay performance under field conditions. Comparison with commercial reference ASFV ELISAs will also be incorporated into future validation studies. Results from these large-scale validation studies will be reported in future publications.

## Figures and Tables

**Figure 1:**
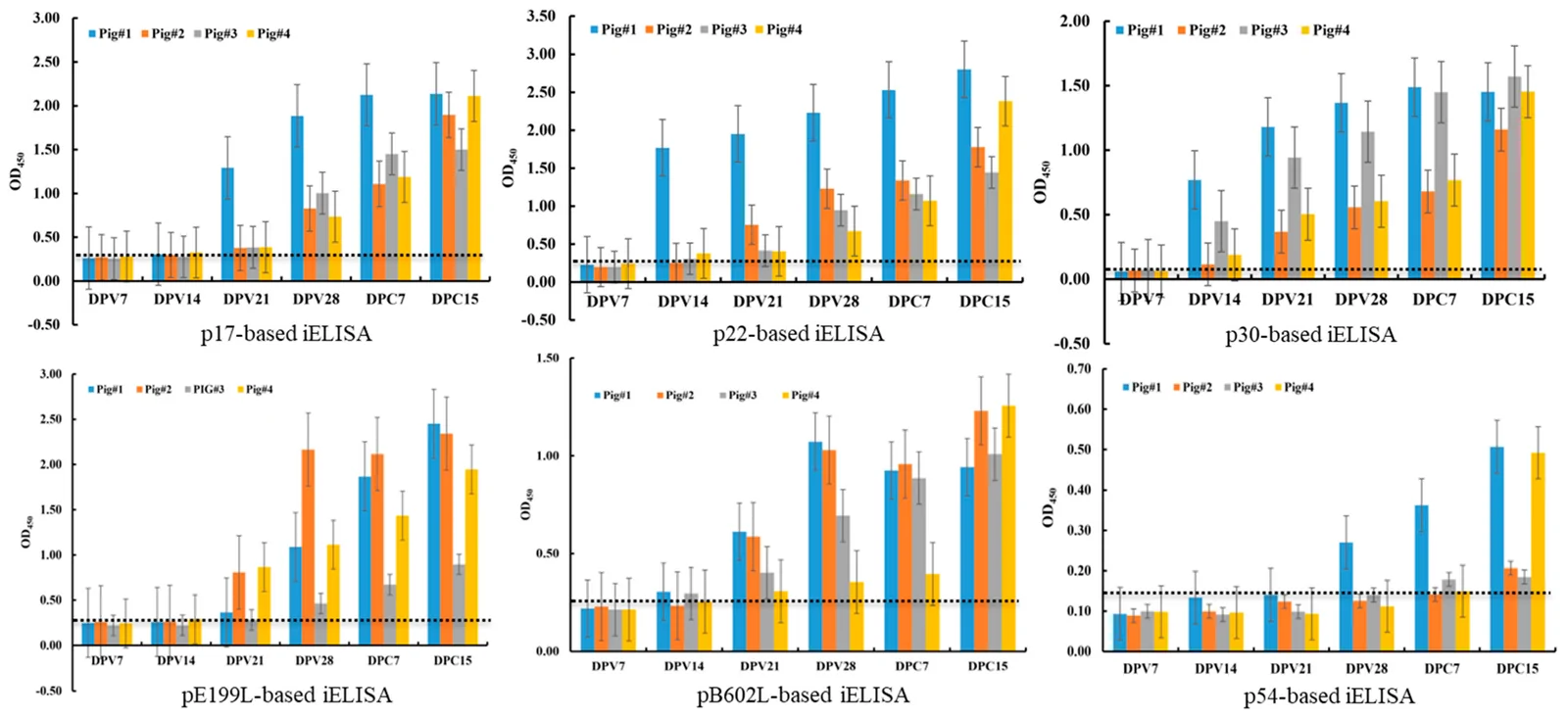
Antibody responses in VNUA-ASFV-LAV2-vaccinated pigs measured by protein-specific iELISAs. Serum samples were derived from pigs vaccinated with VNUA-ASFV-LAV2 (10^3^ HAD_50_/pig) and subsequently challenged with VNUA-ASFV-05L1 (10 HAD_50_/pig). DPV, days post vaccination; DPC, days post challenge. The horizontal dotted line indicates the assay specific cutoff value, defined as the mean OD_450_ of negative-control sera plus three standard deviations (mean + 3SD): p17-based iELISA (0.29), p22-based iELISA (0.28), p30-based iELISA (0.10), pE199L-based iELISA (0.28), pB602L-based iELISA (0.27), p54-based iELISA (0.15).

**Figure 2:**
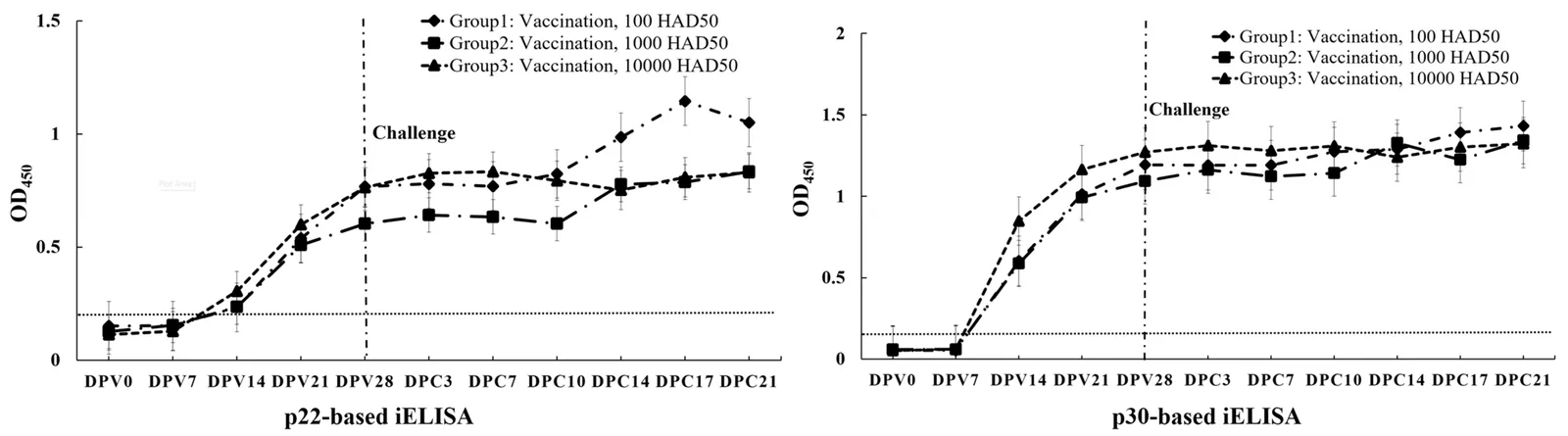
Detection of ASFV-specific antibody responses by p22- and p30-based iELISAs following dose-escalation vaccination and challenge in pigs. Serum samples were collected from pigs vaccinated with VNUA-ASFV-LAV2 at doses of 10^2^, 10^3^, or 10^4^ HAD_50_/pig and subsequently challenged with VNUA-ASFV-05L1 (10 HAD_50_/pig). DPV, days post vaccination; DPC, days post challenge. The horizontal dotted line indicates the assay specific cutoff value, defined as the mean OD_450_ of negative-control sera plus three standard deviations (mean + 3SD): p22-based iELISA (0.21), p30-based iELISA (0.15). Data are expressed as the mean ± standard deviation from independently repeated experiments.

**Figure 3:**
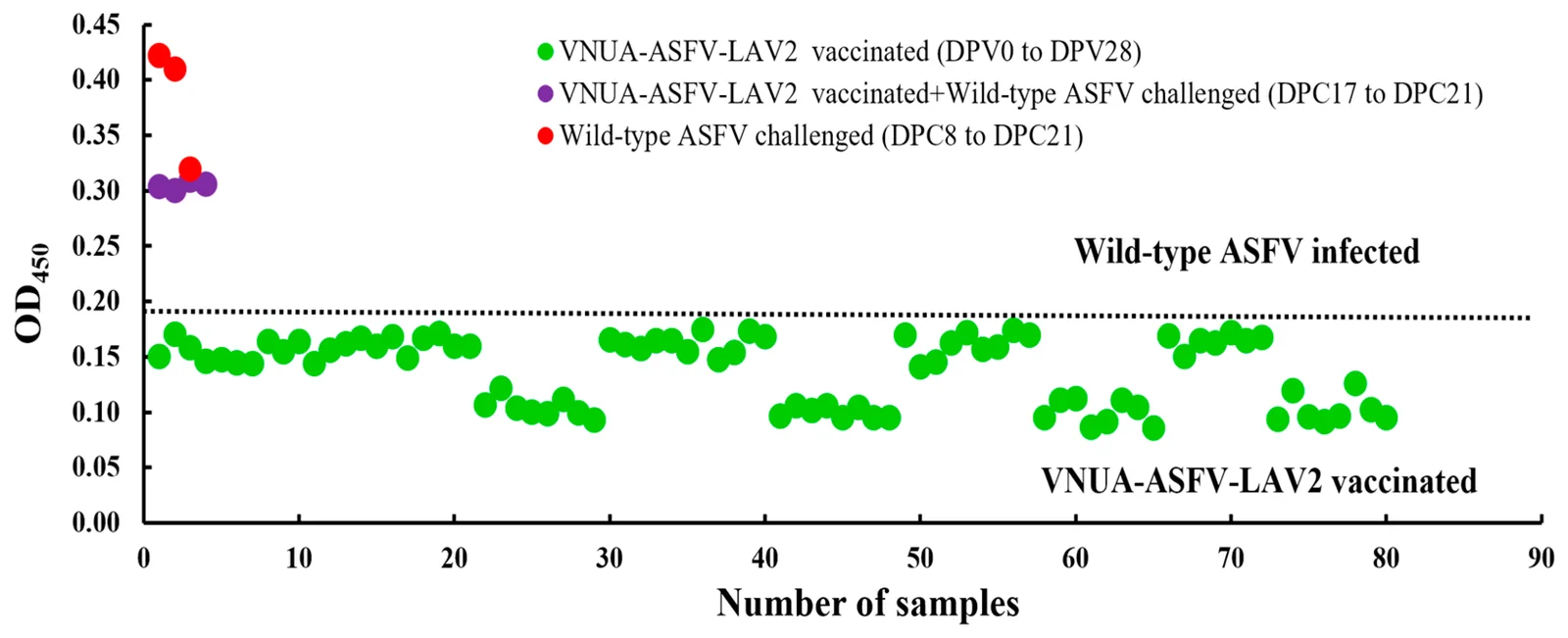
pF317L-based ELISA showed potential to differentiate wild-type ASFV-infected pigs from VNUA-ASFV-LAV2-vaccinated pigs. Serum samples were collected from pigs vaccinated with VNUA-ASFV-LAV2 (10^2^–10^4^ HAD_50_/pig; n = 80), vaccinated and subsequently challenged with VNUA-ASFV-05L1 (10 HAD_50_/pig; n = 4), or infected with wild-type ASFV only (10 HAD_50_/pig; n = 3). The horizontal dotted line indicates the assay cutoff value (0.19), calculated as the mean OD value (0.164; n = 30) of negative-control sera plus three standard deviations (SD; 3 × 0.008).

**Table 1:** ASFV proteins used for indirect ELISA development in this study.

Protein	Gene	Predicted Molecular Weight (kDa)	Functional Category	Reported Function
CD2v	EP402R	~45.3	Outer envelope protein	Hemadsorption, immune modulation, and viral dissemination [26,27,28]
C-type lectin	EP153R	~18.0	Outer envelope protein	Cell adhesion and modulation of host immune responses [28,29]
p72	B646L	~73.2	Major capsid protein	Major capsid protein, highly antigenic and immunogenic, essential for virus replication [28,30]
p54	E183L	~25	Inner envelope protein	Viral entry and intracellular transport [28,31]
p17	D117L	~13.1	Inner envelope protein	Capsid assembly and virion morphogenesis [28,32]
p22	KP177R	~20.2	Inner envelope protein	Host interaction protein involved in endocytosis and signaling [33]
pE199L	E199L	~22.0	Inner envelope protein	Membrane fusion and viral entry [28,34]
p30	CP204L	~30	Inner envelope protein	Early structural protein involved in viral entry [35]
p15 (from pp62)	CP530R	~15.0	Core shell protein	Viral transcription and core shell formation [36]
p35 (from pp62)	CP530R	~35.0	Core shell protein	Core shell assembly and replication [37]
pB602L	B602L	~65.0	Nonstructural protein	Chaperone required for p72 folding [38]
pMGF505-5R	MGF505-5R	~58.6	Nonstructural protein	Viral cell tropism and macrophage replication [39]
pF317L	F317L	~37.6	Uncharacterized protein	Antagonist of innate immune responses, virulence factor [28,40]
pI177L	I177L	~20.4	Uncharacterized protein	Virulence factor [19,20,28]

**Table 2:** Summary of ASFV protein-specific antibody responses in VNUA-ASFV-LAV2-vaccinated pigs evaluated by iELISAs.

Coating Antigens	DPV7	DPV14	DPV21	DPC0	DPC7	DPC15
CD2v (Outer envelope)	-	-	-	-	-	+ (1/4)
C-type lectin (Outer envelope)	-	-	-	-	+ (1/4)	+ (3/4)
p72 (Capsid)	-	-	-	-	-	-
p17 (Inner envelope)	-	-	+ (1/4)	+ (4/4)	+ (4/4)	+ (4/4)
p22 (Inner envelope)	-	+ (2/4)	+ (4/4)	+ (4/4)	+ (4/4)	+ (4/4)
p30 (Inner envelope)	-	+ (3/4)	+ (4/4)	+ (4/4)	+ (4/4)	+ (4/4)
p54 (Inner envelope)	-	-	-	+ (1/4)	+ (1/4)	+ (3/4)
pE199L (Inner envelope)	-	-	+ (2/4)	+ (4/4)	+ (4/4)	+ (4/4)
p15 (Core shell)	-	-	-	-	-	+ (1/4)
p35 (Core shell)	-	-	-	-	-	+ (1/4)
pB602L (Non-structural)	-	-	+ (2/4)	+ (3/4)	+ (3/4)	+ (4/4)
pMGF505-5R (Non-structural)	-	-	-	-	-	-
pF317L (Uncharacterized)	-	-	-	-	-	-
pI177L (Uncharacterized)	-	-	-	-	-	-

Note: “-” = antibody-negative; “+” = antibody-positive; “/” = positive pigs/total pigs tested.

## Data Availability

The datasets used and/or analyzed during the current study are available from the corresponding author upon reasonable request.
